# A novel hexasegmented virus isolated from the phytopathogenic fungus *Verticillium nonalfalfae*

**DOI:** 10.1007/s00705-026-06682-6

**Published:** 2026-06-19

**Authors:** Vanja Miljanić, Sead Sabanadzovic, Nina Aboughanem-Sabanadzovic, Jernej Jakše, Sebastjan Radišek, Nataša Štajner

**Affiliations:** 1 Department of Agronomy, Biotechnical Faculty, University of Ljubljana, Ljubljana, 1000 Slovenia; 2 Department of Agricultural Science and Plant Protection, Mississippi State University, Mississippi State, MS 39762 USA; 3 Biocomputing and Biotechnology, Institute for Genomics, Mississippi State University, Mississippi State, MS 39762 USA; 4 Plant Protection Department, Slovenian Institute of Hop Research and Brewing, Žalec, 3310 Slovenia

## Abstract

In this study, we describe a novel virus, tentatively named Verticillium nonalfalfae virus M (VnaVM), identified in the phytopathogenic fungus *Verticillium nonalfalfae* isolated from hop (*Humulus lupulus* L.). The VnaVM genome consists of six double-stranded RNA (dsRNA) segments ranging from 1051 to 2401 bp. DsRNAs 1–3 encode an RNA-dependent RNA polymerase (RdRp), a hypothetical protein and a methyltransferase (MTR), respectively, while dsRNA6 encodes a proline-alanine-serine-rich protein (PASrp). These four proteins share less than 58% amino acid identity with homologs encoded by viruses in the family *Polymycoviridae*. Products encoded by dsRNAs 4 and 5 show no similarity to known proteins, and their roles in the viral life cycle are unknown. Phylogenetic analysis of RdRp amino acid sequences, together with genome organization and pairwise identity values, supports classifying VnaVM as a representative of a novel species within the genus *Multimycovirus* (family *Polymycoviridae*). To our knowledge, this is the first report of a polymycovirid infection in a member of the genus *Verticillium*.

**Supplementary Information:**

The online version contains supplementary material available at 10.1007/s00705-026-06682-6.

Polymycovirids, viruses belonging to the *Polymycoviridae* family, are a group of recently described mycoviruses. The first polymycovirid was discovered in the human pathogenic fungus *Aspergillus fumigatus* [1]. Since then, polymycoviruses have also been identified in a range of plant- [2–6] and insect-associated fungi [7–10]. These viruses are notable for their unusual genomic structure, which typically comprises four to eight dsRNA segments, each containing one, or rarely two, putative open reading frames (ORFs) flanked by 5′- and 3′-untranslated regions (UTRs). All known polymycovirids share a “core” of four conserved genomic segments coding for RNA-dependent RNA polymerase (RdRp), a hypothetical cysteine-rich protein, putative methyltransferase (MTR), and a proline-alanine-serine-rich protein (PASrp) [11]. The family *Polymycoviridae*, officially recognized in 2020 as a monogeneric family, has recently been reorganized and expanded to include 28 species classified into three genera: *Polymycovirus*, *Plurimycovirus* and *Multimycovirus* [12, 13]. Evolutionarily, polymycovirids are distantly related to hadakavirids (family *Hadakaviridae*), both currently classified in the order *Xenadelphovirales*, class *Mycopleornaviricetes* [12, 13]. Some polymycovirids have been shown to alter various host features, such as growth, pigmentation, sporulation, changes in virulence, fungicide susceptibility, and metabolic pathways [7, 14–16].

Verticillium wilt, caused by ascomycete filamentous fungi in the genus *Verticillium*, is among the most devastating plant vascular fungal diseases worldwide. Ten species are currently recognized in the genus *Verticillium* sensu stricto (*V. albo-atrum*, *V. alfalfae*, *V. dahliae*, *V. isaacii*, *V. klebahnii*, *V. longisporum*, *V. nonalfalfae*, *V. nubilum*, *V. tricorpus*, and *V. zaregamsianum*) [17]. These soilborne fungi can persist in soil for several years without a host by producing melanized resting structures [18]. Following host-induced germination of these resting structures, the fungus penetrates the root system and subsequently colonizes the xylem, resulting in wilting and, in severe cases, complete dieback [19]. To date, a total of seven mycoviruses have been reported from *Verticillium* spp.: Verticillium dahliae ormycovirus 1 (VdOMV1) and VdOMV2 (family *Ormycoviridae*), Verticillium dahliae chrysovirus 1 (VdCV1; family *Chrysoviridae*), Verticillium dahliae partitivirus 1 (VdPV1; family *Partitiviridae*), Verticillium dahliae magoulivirus 1 (VdMoV1; family *Botourmiaviridae*), Verticillium dahliae RNA virus 1 (VdRV1; unassigned non-segmented + RNA virus), and Verticillium albo-atrum partitivirus 1 (VaaPV1; family *Partitiviridae*) [20–25].

Hop (*Humulus lupulus* L.) is one of the most susceptible hosts to *V. nonalfalfae*. Highly virulent or lethal pathotypes spread rapidly throughout hop plantations, resulting in significant yield losses and long-term impacts on hop production [26]. In this study, we report the molecular characterization of the novel hexasegmented virus from the phytopathogenic fungus *V. nonalfalfae*, tentatively named Verticillium nonalfalfae virus M (VnaVM).

The source material for dsRNA isolation was *V. nonalfalfae* isolate 274VD, recovered from an infected hop plant in Slovenia in 2024. Fungal mycelia were harvested from a pure culture grown at room temperature on a cellophane membrane overlaid on the surface of ½ Czapek Dox plate (supplemented with 1 g/l of malt extract, 1 g/l of yeast extract, 1 g/l of peptone, and 15 g/l of agar). DsRNA was extracted from fungal mycelium using cellulose C6288 (Sigma-Aldrich, St. Louis, MO, USA), following the method described by Mokhtari and Ali [27] with minor modifications. The nature of the dsRNA was confirmed by digestion of the extracts with DNase I (New England BioLabs) and S1 nuclease (Thermo Fisher Scientific). The purified dsRNAs were analyzed in a 1% (wt/vol) agarose gel and visualized under UV light after staining with 0.1 µg/mL ethidium bromide. Six dsRNA segments ranging from 1 to 2.4 kb were observed. The library was constructed using the Ion Total RNA-Seq Kit v2 (Thermo Fisher Scientific) according to the manufacturer’s instructions. The yield and size distribution of the amplified cDNA library were determined using the Agilent 2100 Bioanalyzer (Agilent Technologies). Sequencing was performed on the Ion GeneStudio S5 Prime System (Thermo Fisher Scientific). Adapter sequences were removed from raw reads before processing with bioinformatics tools CLC Genomic Workbench and Genomics Server (Qiagen). The Basic Local Alignment Search Tool (BLAST) of the National Center for Biotechnology Information (NCBI) was used for sequence comparisons. Potential open reading frames (ORFs) and putative conserved domains were predicted using ORF Finder and the Conserved Domain Database (CDD) of NCBI, respectively. Proteins with no matches in CDD, were further investigated using HHPred for remote homology and structure prediction via pairwise comparison of profile hidden Markov models (HMMs) [28]. Phylogenetic analysis was conducted with the IQ-Tree v.3.0.1 [29] on amino acid sequences of RdRp aligned with MAFFT v.7 [30] using the best-fit model Q.PFAM + F+I+G4 identified with ModelFinder [31]. The tree was visualized with iTOL v7 [32].

The complete sequences of dsRNA1-6 were obtained by rapid amplification of cDNA ends (RACE) using the SMART™ RACE cDNA Amplification Kit (Takara). All amplified fragments were purified, cloned, and Sanger sequenced. The complete sequence of each dsRNA segment was determined by sequencing at least three independent clones. Primer sets used in this study are detailed in Supplementary Table 1. The six dsRNAs are deposited in the GenBank database under the accession numbers PX960521-PX960526.

A total of six dsRNA segments were extracted from *V. nonalfalfae* isolate 274VD (Fig. 1 A) and visualized by agarose gel electrophoresis (Fig. 1B). Each dsRNA segment contained a single open reading frame on the coding strand flanked by UTRs (Fig. 1C). The 5′-UTRs of the coding strands of dsRNAs 1–6 range from 30 to 117 nt in length and share a highly conserved 30-nt sequence (ACAUGGGGGAACAAAAYHWUAUAWMYKYRC) (Supplementary Fig. 1A). The 3′-UTRs vary from 64 to 299 nt in length and lack extensive conserved elements. Secondary structure prediction revealed that the 5′ and 3′ terminal regions of all six dsRNAs fold into stem–loop structures, as exemplified by dsRNA1 (Supplementary Fig. 1B).

VnaVM dsRNA1 contains one large ORF1 (nt 31-2337), which encodes a protein of 768 amino acids (aa) with an estimated molecular mass (*Mr*) of 84.07 kDa. The 84 K protein shares high amino acid identity with RdRps encoded by members of the family *Polymycoviridae*, particularly with that encoded by Exserohilum turcicum polymycovirus 1 (XBY85583.1) and Setosphaeria turcica polymycovirus 1 (UMZ55610.1) (57.96%, query cover = 100%, e-value = 0.0). VnaVM RdRp contains the six conserved motifs, including the characteristic tetrapeptide GDNQ in motif VI (Fig. 2A). This tetrapeptide is also found in members of the mycoviral families *Polymycoviridae* and *Hadakaviridae*, as well as in some (-)RNA viruses belonging to the order *Mononegavirales* [33].

VnaVM dsRNA2 contains a single ORF (ORF2, nt 79-2172) that encodes a hypothetical protein of 697 aa (*Mr* 74.03 kDa) of unknown function, which shares amino acid identity content (25–48%) with the corresponding proteins of polymycovirids and Hadaka virus 1.

The third dsRNA of VnaVM is also monocistronic, with a coding region spanning nucleotides 47 to 1900 and encoding a protein of 617 aa (*Mr* 66.56 kDa). The ORF3-encoded protein shows the highest identity of ~ 48% with homologs encoded by dsRNA3 of Alternaria alternata polymycovirus 1 (QVK45098.1) and Plasmopara viticola lesion associated polymycovirus 1 (QHG11068.1) (query cover = 89%; e-value = 7e-170). CDD searches indicate that the ORF3 protein contains a conserved RsmD superfamily domain from amino acid positions 129 to 207 (COG0742; e-value = 1.21e-06), suggesting that it is a SAM-dependent methyltransferase.

The sixth dsRNA contains a single ORF (ORF6, nt 118–906) encoding a 262-aa product (*Mr* 28.03 kDa) relatively rich in proline (8.02%), alanine (10.68%), and serine (8.02%). A BLASTp search showed that the ORF6 protein sequence shares 56.70% identity with PASrps of Beauveria bassiana polymycovirus 4 (QRF54816.1), Beauveria bassiana polymycovirus 4 − 2 (UXC94315.1), and Lecanicillium aphanocladii polymycovirus 1 (XUP88367.1) (query cover = 100%; e-value = 1e-96). It contains a Pfam-annotated capsid protein domain, spanning amino acid residues 4-259, related to that of Colletotrichum camelliae filamentous virus 1 (PF25660, CcFV1_CP, e-value = 1.2e-95), indicating that it likely represents the capsid protein of the virus.

DsRNA4 and dsRNA5 each contain a single ORF (nt 97-1113 and 106–1101, respectively) encoding proteins with predicted molecular masses of 36.26 kDa and 35.72 kDa, respectively, with no significant similarity to known proteins.

Phylogenetic analysis of the VnaVM RdRp, along with homologous proteins encoded by recognized and putative polymycovirids and by hadaka virus 1, suggests that VnaVM is a new member of the family *Polymycoviridae*. Based on the RdRp tree topology, VnaVM is evolutionarily closest to Trichoderma barbatum polymycovirus 1 and groups with other members of the newly established genus *Multimycovirus* (Fig. 2B). Similar results were obtained in phylogenetic analysis of the amino acid sequences of MTR and PASrp (not shown).

The phylogenetic position and genomic characteristics of VnaVM indicate its affiliation with the genus *Multimycovirus* in the family *Polymycoviridae*. Furthermore, VnaVM RdRp shares less than 58% sequence identity with homologs encoded by members of previously described species, which is below the proposed species demarcation threshold in the family (> 70% RdRp aa identity), supporting its classification as a novel member of this genus. To our knowledge, this is the first report of a polymycovirus infection in any *Verticillium* species. Given that some polymycoviruses influence the phenotype of their fungal hosts, ongoing research focuses on assessing the impact of VnaVM on host virulence.

**Fig. 1:**
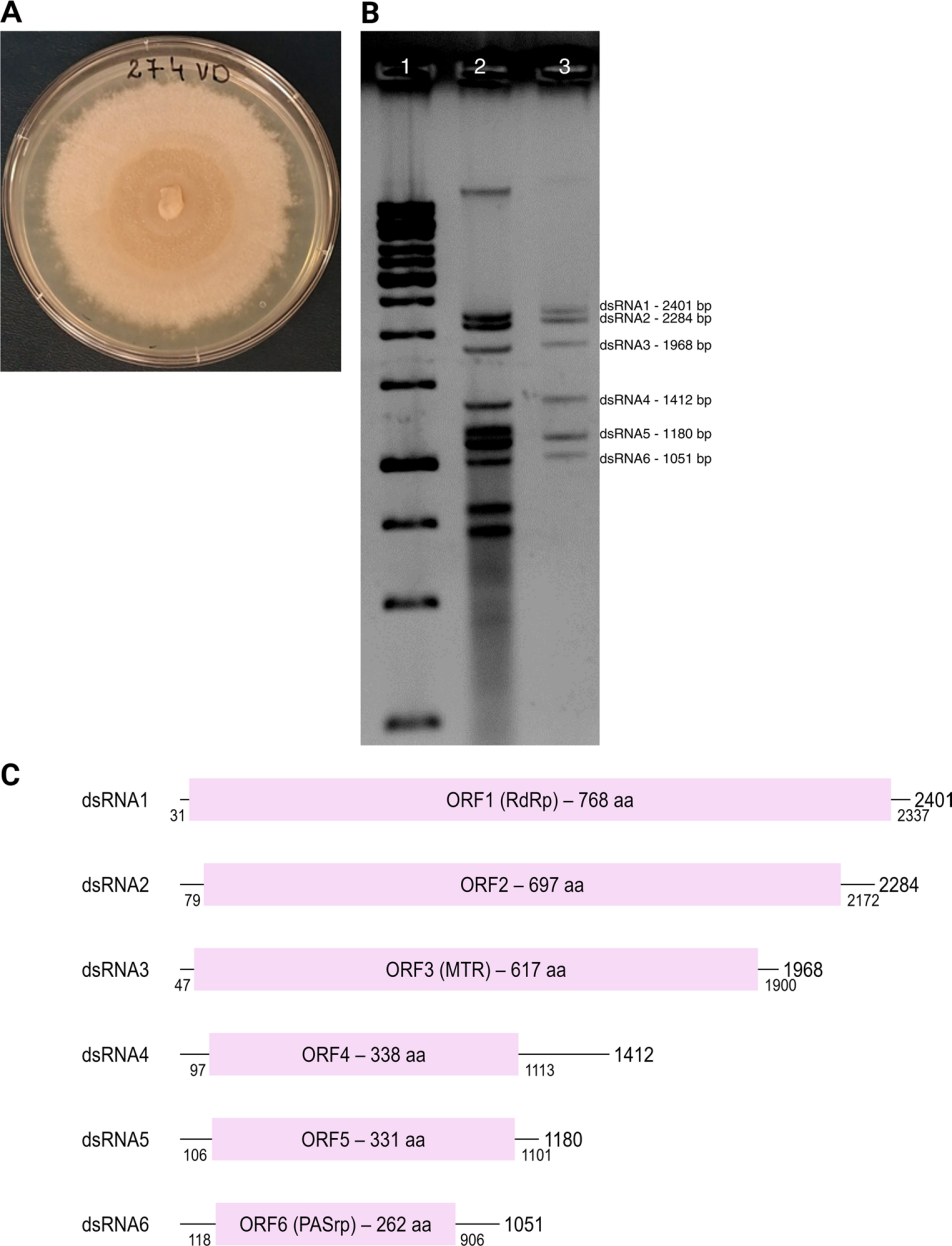
(**A**) Fungus *V. nonalfalfae* isolate 274VD. (**B**) dsRNA was electrophoresed in a 1% agarose gel. Lane 1, DNA ladder; lane 2, dsRNA without S1 nuclease and DNase I treatment; lane 3, dsRNA treated with S1 nuclease and DNase I. (**C**) Schematic representation of the genome organization of VnaVM RNAs 1 to 6. Pink boxes indicate ORFs, and black lines indicate UTRs

**Fig. 2:**
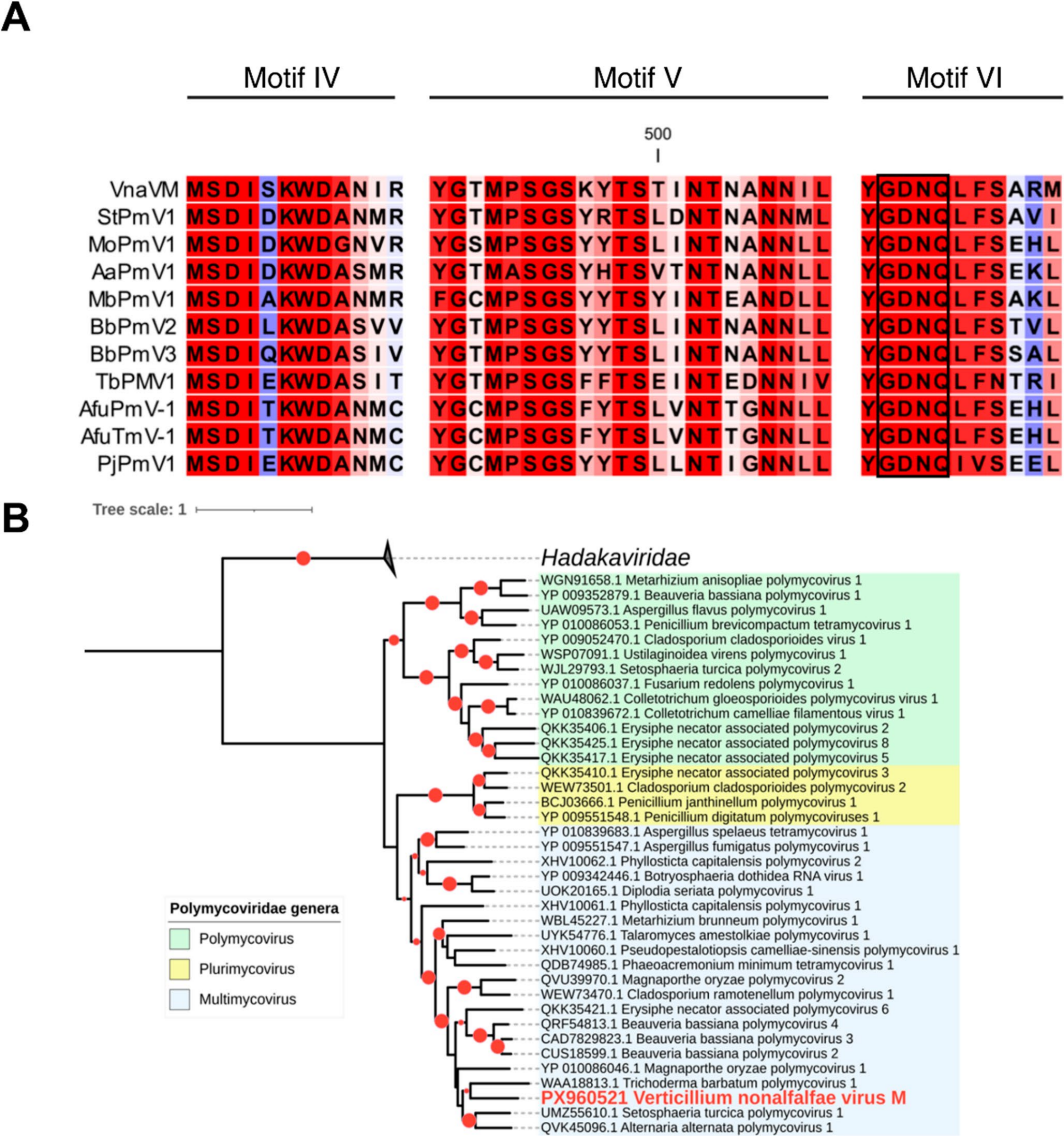
(**A**) Amino acid sequence alignment of RdRp motifs IV-VI of Verticillium nonalfalfae virus M (VnaVM) and other members of the family *Polymycoviridae*. StPmV1, Setosphaeria turcica polymycovirus 1; MoPmV1, Magnaporthe oryzae polymycovirus 1; AaPmV1, Alternaria alternata polymycovirus 1; MbPmV1, Metarhizium brunneum polymycovirus 1; BbPmV2, Beauveria bassiana polymycovirus 2; BbPmV3, Beauveria bassiana polymycovirus 3; TbPMV1, Trichoderma barbatum polymycovirus 1; AfuPmV-1, Aspergillus fumigatus polymycovirus 1; AfuTmV-1, Aspergillus fumigatus tetramycovirus 1; PjPmV1, Penicillium janthinellum polymycovirus 1. (**B**) Maximum-likelihood phylogenetic tree showing the relationships of VnaVM with members of the two families in the order *Xenadelphovirales*. VnaVM groups with members of the genus *Multimycovirus* (clade shaded blue) in the family *Polymycoviridae*. Clades corresponding to the other two genera in this family, *Polymycovirus* and *Plurimycovirus*, are shaded in green and yellow, respectively. The tree was constructed on the MAFFT-aligned [30] RdRp amino acid sequences using IQ-TREE v 3.0.1 [29]., using the best-fit model “Q.PFAM + F+I+G4” according to BIC, as estimated by ModelFinder [31]. The tree was visualized with iTOL v7 [32]. The GenBank accession numbers of RdRp amino acid sequences used for analysis along with virus names are indicated at the branch tips. Red dots at a branching point indicate statistical support > 90%, with the size corresponding to the bootstrap value. The clade representing the second family in the order, *Hadakaviridae*, is collapsed

## Supplementary Information

Below is the link to the electronic supplementary material.

Supplementary Material 1

## Data Availability

No datasets were generated or analysed during the current study.
