# Supplementary material for: A novel hexasegmented virus isolated from the phytopathogenic fungus Verticillium nonalfalfae
Source: Arch Virol. 2026 Jun 19;171(7):216. doi: 10.1007/s00705-026-06682-6 (PMC13282248; doi:10.1007/s00705-026-06682-6)
Supplement: Supplementary file 1 — Supplementary Material 1 [file 705_2026_6682_MOESM1_ESM.docx]

**Supplementary Material**

A novel hexasegmented virus isolated from the phytopathogenic fungus *Verticillium nonalfalfae*


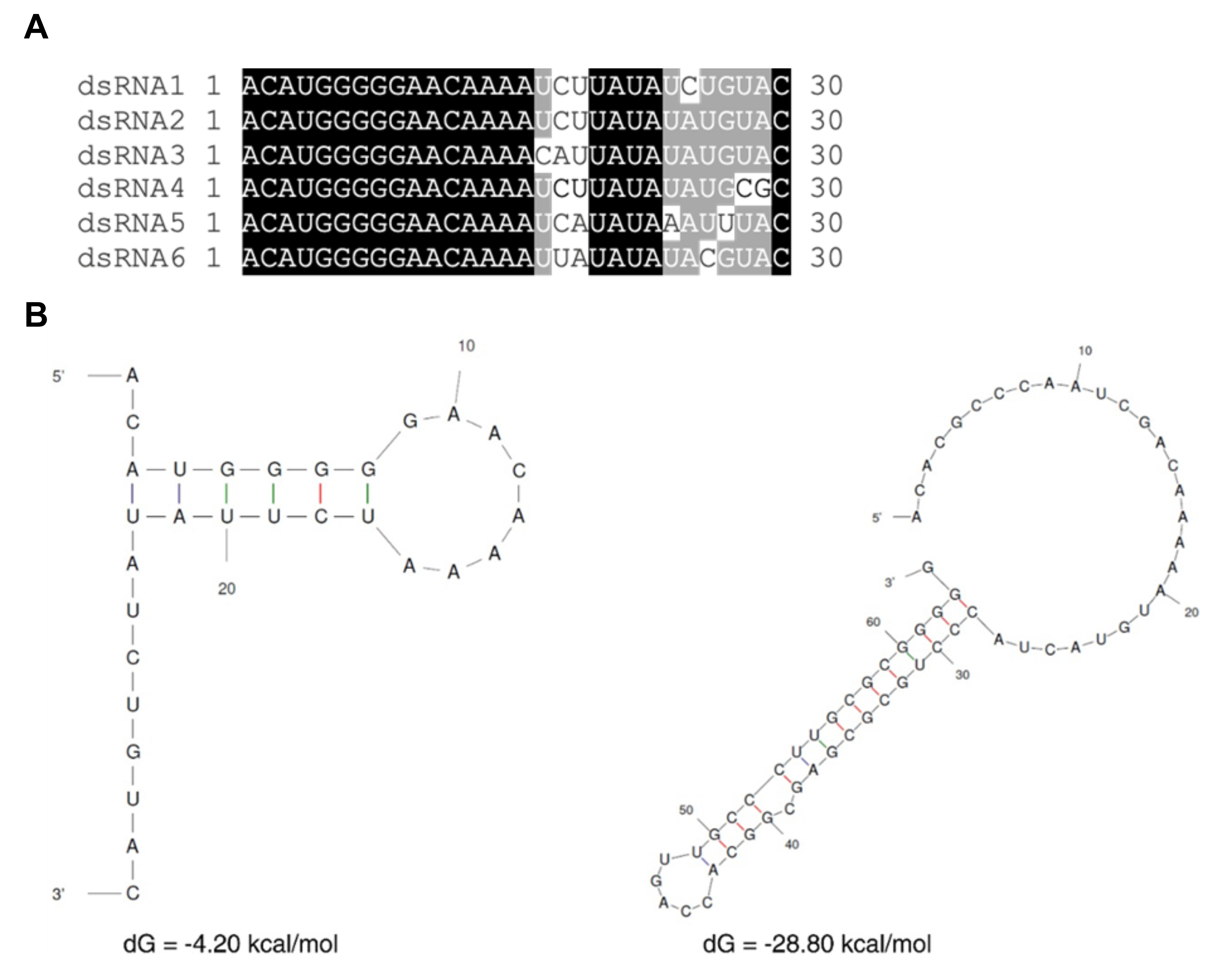


**Supplementary Figure 1: (A)** Alignment of the initial 30 nt of the Verticillium nonalfalfae virus M six genomic segments shows high conservation of the viral 5’terminal sequences. Black-shaded nucleotides are universally conserved across all dsRNA molecules, while gray-shaded residues are conserved in five of six genomic sequences. **(B)** The predicted secondary structures with the lowest energy are shown for the 5′ (left) and 3′ (right) termini of RNA1.

| **Primer Name** | **Sequence (5′ → 3′)** |
| --- | --- |
| 5′_dsRNA1 | GATTACGCCAAGCTTCGGTCGATGATGGAATTGGTGAGCCCCT |
| 3′_dsRNA1 | GATTACGCCAAGCTTTGACAACCAGCTCTTTTCCGCCCGC |
| 5′_dsRNA2 | GATTACGCCAAGCTTCGGTGGACACAGATGGGGCTAATAGGGC |
| 3′_dsRNA2 | GATTACGCCAAGCTTGCGAGCGTTGCCAGACTTTCTCCCG |
| 5′_dsRNA3 | GATTACGCCAAGCTTTCGGAGTAAAGGGCGTCGATGGTGCAAA |
| 3′_dsRNA3 | GATTACGCCAAGCTTCTCGACCGCCTTCTCTCCTGTGCCA |
| 5′_dsRNA4 | GATTACGCCAAGCTTAGTCACACTGAAACTGTCGTAGCCGCGC |
| 3′_dsRNA4 | GATTACGCCAAGCTTCTGGGGCAGGGCTGTATGTCATCGC |
| 5′_dsRNA5 | GATTACGCCAAGCTTGATCGGACAGCAGACCAAGATCGCGAGC |
| 3′_dsRNA5 | GATTACGCCAAGCTTAGGCGCAACAACAGCTTCTTCCCGG |
| 5′_dsRNA6 | GATTACGCCAAGCTTGTGGGGTTGCGGTTGATCTCCTCCTTGA |
| 3′_dsRNA6 | GATTACGCCAAGCTTAGGAGATCAACCGCAACCCCACCCT |

**Supplementary Table 1:** List of primers used for 5’- and 3’- RACE. Each has 15 bp overlaps (GATTACGCCAAGCTT) with the vector at their 5’ ends to facilitate In-Fusion cloning according to the manufacturer’s instructions.
